# Will Earth's next supercontinent assemble through the closure of the Pacific Ocean?

**DOI:** 10.1093/nsr/nwac205

**Published:** 2022-09-28

**Authors:** Chuan Huang, Zheng-Xiang Li, Nan Zhang

**Affiliations:** Earth Dynamics Research Group, The Institute for Geoscience Research (TIGeR), School of Earth and Planetary Sciences, Curtin University, Perth 6845, Australia; Key Laboratory of Orogenic Belts and Crustal Evolution, School of Earth and Space Sciences, Peking University, Beijing 100871, China; Earth Dynamics Research Group, The Institute for Geoscience Research (TIGeR), School of Earth and Planetary Sciences, Curtin University, Perth 6845, Australia; Earth Dynamics Research Group, The Institute for Geoscience Research (TIGeR), School of Earth and Planetary Sciences, Curtin University, Perth 6845, Australia; Key Laboratory of Orogenic Belts and Crustal Evolution, School of Earth and Space Sciences, Peking University, Beijing 100871, China

**Keywords:** yield stress, oceanic lithosphere, introversion, extroversion, supercontinent cycle

## Abstract

Earth's known supercontinents are believed to have formed in vastly different ways, with two endmembers being introversion and extroversion. The former involves the closure of the internal oceans formed during the break-up of the previous supercontinent, whereas the latter involves the closure of the previous external superocean. However, it is unclear what caused such diverging behavior of supercontinent cycles that involved first-order interaction between subducting tectonic plates and the mantle. Here we address this question through 4D geodynamic modeling using realistic tectonic set-ups. Our results show that the strength of the oceanic lithosphere plays a critical role in determining the assembly path of a supercontinent. We found that high oceanic lithospheric strength leads to introversion assembly, whereas lower strength leads to extroversion assembly. A theoretically estimated reduction in oceanic crustal thickness, and thus its strength, during Earth's secular cooling indicates that introversion was only possible for the Precambrian time when the oceanic lithosphere was stronger, thus predicting the assembling of the next supercontinent Amasia through the closure of the Pacific Ocean instead of the Indian-Atlantic oceans. Our work provides a new understanding of the secular evolution of plate tectonics and geodynamics as the Earth cooled.

## Teaser

Geodynamic modeling shows that the yield strength of the oceanic lithosphere determins the assembly path of a supercontinent. The assembling of the next supercontinent Amasia through the closure of the Pacific Ocean is predicted.

**A comment on this article is 'Comment on 'Will Earth's next supercontinent assemble through the closure of the Pacific Ocean?' by Steinberger (https://doi.org/10.1093/nsr/nwac253)**.

## INTRODUCTION

**Figure 1.**
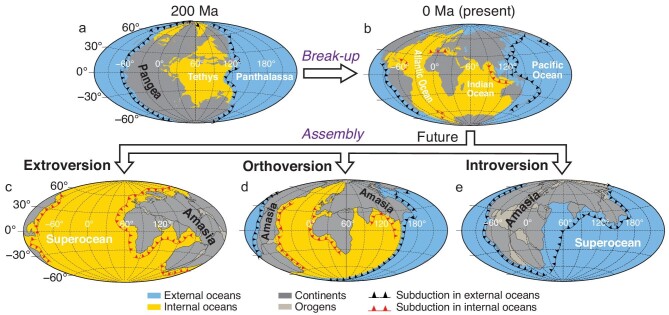
Cartoon illustrating three possible ways of assembling the future supercontinent Amasia from the break-up of Pangaea (a and b): (c) extroversion [4], (d) orthoversion [13] and (e) introversion [11].

A primary feature of Earth's tectonic evolution since around 2 billion years ago (Ga) is the supercontinent cycle [1–3], featuring a cyclical assembly and dispersal of major continents with a periodicity of around 600 million years (Myrs) [4]. Two endmember forms of supercontinent assembly have been proposed [5]. Introversion assembly involves the closure of internal oceans created during the break-up of the previous supercontinent, whereas extroversion assembly involves the closure of the external superocean surrounding the previous supercontinent [6] (Fig. 1). Of the three known supercontinents, the oldest one, Nuna/Columbia (1.6–1.3 Ga) [7], could be Earth's first supercontinent [8], and its assembly therefore does not involve the introversion/extroversion processes. However, how the two younger supercontinents, Rodinia (0.9–0.7 Ga) [9] and Pangea (0.32–0.17 Ga) [10], formed remains controversial. Li *et al*. [11] speculated that Rodinia formed through introversion whereas Pangea formed through extroversion, but others argued otherwise [6,12]. To complicate the matter further, Mitchell *et al*. [13] proposed that a supercontinent could assemble through orthoversion by closing the minor oceans between the continents when they gather along the girdle of subduction ∼90° away from the center of the previous supercontinent (Fig. 1d). Understanding how each supercontinent assembled and the controlling forces behind it is important not only for understanding how the plate tectonics system interacts with mantle dynamics in an evolving Earth, but also for predicting if the next supercontinent, dubbed Amasia [14], will form through the closure of the Pacific Ocean, the Atlantic Ocean or neither [11,13,14].

The style of supercontinent assembly, and that of plate tectonics in general, is likely linked to both the properties of tectonic plates and their interactions with the Earth's mantle in the context of a secularly cooling Earth. Here we use 4D geodynamic modeling with realistic plate and mantle parameter settings to examine if factors like lithospheric strength, Earth's secular cooling, and the volume and density of the lower-mantle thermo-chemical piles as presented by the two present-day large low shear velocity provinces (LLSVPs) [15,16], play a critical role in determining whether a supercontinent is assembled through introversion, extroversion or orthoversion (Fig. 1). With our modeling results, we speculate if the next supercontinent will likely assemble through the closure of the Pacific Ocean or the Indo-Atlantic oceans.

## MODEL SETTINGS

We set our dynamic models on the coupling processes between Earth-like plate tectonics and mantle convection over a complete supercontinent cycle. Each model starts from the assembly of the mother supercontinent (supercontinent-1) to establish the initial condition (see Methods in Supplementary Data) for the modeling. It then proceeds to the break-up of the mother supercontinent as the beginning of a new supercontinent cycle, and finishes at the formation of the daughter supercontinent (supercontinent-2). Our model set-up features the following characteristics. (i) Continents are defined as chemically distinct regions that are buoyant and move self-consistently over the sub-lithospheric mantle [17]. (ii) The oceanic lithosphere deforms in a pseudoplastic fashion when the local stress is larger than its yield stress, such that Earth-like ocean–ocean (two-sided subduction due to the limitation of pseudoplastic approximation of the oceanic lithosphere) or ocean–continent subduction (one-sided), driven by dynamic processes, can initiate [17,18]. (iii) Self-generation of ocean–continent subduction is made possible by adding low viscosity (weak) zones [19,20] along the continental margins when the nearby oceanic lithosphere is older than 200 Myrs, thus the oceanic plates’ negative buoyancy from cooling can initiate subduction. Such weak zones are removed when oceanic crust younger than 10 Myrs reaches the subduction zone, which is expected to slow or even jam the subduction [17] (see Methods in Supplementary Data). (iv) A dense chemical layer in the lower mantle above the core-mantle boundary (CMB), sourced from either the remanent of the primordial magma ocean [21] and/or the subducted oceanic slabs [22,23], is implanted at the beginning of the modeling to simulate the formation and evolution of the LLSVPs. (v) Weak orogens are automatically generated when two adjacent continents are joined together through the closure of the ocean between them, which not only prevent the colliding continents from becoming a single large craton, but also play a guiding role for the future break-up of the supercontinent [24] (see Methods in Supplementary Data). Collectively, these settings (Table S1 in Supplementary Data) enable our models to simulate the Earth-like mantle–plate coupling process to the first order [17,18,25].

## RESULTS

### Strength of the oceanic lithosphere and supercontinent assembly

**Figure 2.**
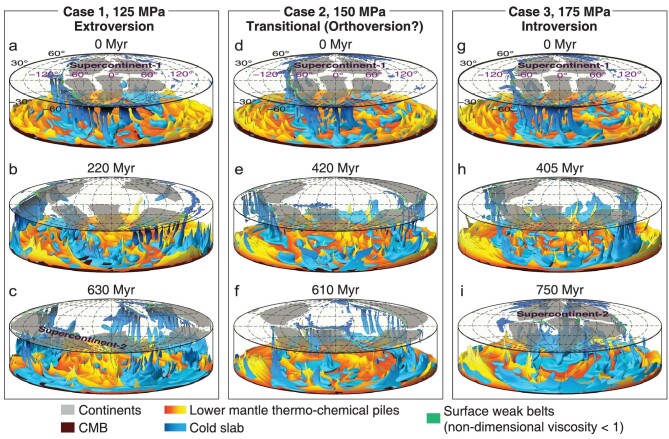
Modeling results of a full supercontinent cycle with different oceanic lithospheric yield stresses. (a–c) Evolutionary snapshots for the extroversion supercontinent assembly (Case 1) at (a) 0 Myrs, (b) 220 Myrs and (c) 630 Myrs, with a lowest yield stress of 125 MPa. (d–f) The transitional (orthoversion?) supercontinent assembly (Case 2) with intermediate yield stress of 150 MPa. (g–i) The introversion supercontinent assembly (Case 3) with a highest yield stress of 175 MPa. All other parameters are the same for the three models. The subducting cold slabs are shown as –0.1 isocontours of the mantle's non-dimensional residual temperature (–0.05 for the top 300 km). All the calculations are performed in 3D spherical geometry, and the results are first unwrapped into cartesian coordinates and then converted into Hammer projections at each depth for easier visualization.

With carefully chosen initial temperature field (see Methods in Supplementary Data), we first run three cases to examine the influence of yield stress of the oceanic lithosphere on how a supercontinent is assembled, with the yield stress set at 125 MPa (Case 1), 150 MPa (Case 2) and 175 MPa (Case 3), respectively, all within the previously suggested range [26] (see Methods in Supplementary Data). The simplified yield stress profiles for the oceanic lithosphere allow for the modeling of geodynamic processes with reasonable proximations of the lithospheric strength [18,27]. Continents in Case 1 first disperse during the break-up of the initial supercontinent-1 and become scattered along a great circle inside the retreating subduction girdle by ∼220 Myrs (Fig. 2a and b; Fig. S1a and b in Supplementary Data). The maximum root-mean-square (simplified as mean hereafter) velocity of the continents reaches its peak soon after the continents drift pass the great circle to the other side of the globe at ∼280 Myrs (Fig. S2a). The continents then start to converge at the opposite hemisphere with reducing mean velocity (Fig. 2b and c; Figs S1b and c and S2a) as if they are pulled by the retreating, first enlarging, and then shrinking subduction girdle until the final assembly of the new supercontinent-2 (Fig. S1a–c). By that stage, the previous superocean-1 surrounding supercontinent-1 (Fig. S1a) is entirely consumed, whereas a new subduction girdle forms along the shared margin between the newly formed supercontinent-2 and superocean-2 that has grown from the early internal ocean (Fig. S1b and c) in a clear case of extroversion supercontinent assembly (Movie S1) [11].

Case 2 repeats the same procedure as Case 1 but a yield strength of 150 MPa is applied to the oceanic lithosphere. We find that the breaking-away continents still first spread following the expanding (retreating) subduction girdle (Fig. 2d and e; Fig. S1d and e), but then stop spreading further once the maximum dimension of the internal ocean reaches ∼180° by ∼420 Myrs (Fig. S1e). The continents remain relatively stable in such a girdle configuration for over 200 Myrs, with subduction occurring around both the external and internal oceans (Fig. 2e and f; Fig. S1e and f). The continents are almost connected, mimicking an orthoversion assembly of supercontinent-2 (Movie S2) [13].

Case 3 has the highest yield stress value of 175 MPa for the oceanic lithosphere. In this case, the break-away continents first spread following the retreating subduction girdle (Fig. 2g and h; Fig. S1g and h). However, the continental dispersion stops at ca. 405 Myrs when the maximum dimension of the growing internal ocean reaches ∼180° (Fig. S1h). After that, the internal ocean starts to shrink as the continents start to move back toward the location of the original supercontinent until the internal ocean is closed and the new supercontinent-2 is assembled

at about the same location as its predecessor, constituting an introversion assembly (Fig. 2h and i; Fig. S1h and i and Movie S3) [6,11].

These results indicate that the strength of the oceanic lithosphere can determine how a supercontinent is assembled. Once the break-away continents are dispersed along a girdle constrained by two subduction systems around both the shrinking external ocean and the growing internal ocean, how the next supercontinent is assembled appears to be strongly influenced by the competing pulling power of subduction systems in the two oceans. A low oceanic yield strength (Case 1) leads to lower effective viscosity (see the definition in Methods of Supplementary Data) in the oceanic lithosphere and makes subduction of the oceanic lithosphere easier, facilitating the formation of strong degree-1 mantle convection power (Fig. S3) with a super-downwelling being formed under the initial external superocean-1 (Fig. 2b and c; Movie S1), thus, an extroversion assembly of supercontinent-2 over such a super-downwelling [28]. In such a case, the continents disperse the fastest during the assembly (Fig. S4a) and reach the farthest distance away from that of supercontinent-1 when new subduction starts to form in the internal ocean. Also, the subduction rate in the external ocean is significantly higher in this case compared to the other cases at ∼220–350 Myrs (Fig. S4b). In such a case, it is most difficult for subduction in the internal ocean to drag the continents back. With a higher strength for the oceanic lithosphere (Case 3, Fig. 2g–i), the effective viscosity of the oceanic lithosphere becomes higher and no prominent super-downwelling is formed under the external ocean as in Case 1. Instead, the continents are pulled back by the stronger subduction system in the internal ocean toward the final stage of the daughter supercontinent-2 assembly (Fig. 2h and i; Fig. S1h and i and S4b). With an intermediate oceanic lithospheric strength (Case 2; Fig. 2d–f), the power of subduction in the internal and external oceans becomes comparable by ∼550 Myrs (Fig. S4b), leading to the formation of a persistent and stable degree-2 mantle convection power (Fig. 2e and f; Fig. S3) and a potential supercontinent formed above the subduction girdle defined by the two subduction systems (Fig. S1f).

### Possible effects of the lower-mantle thermo-chemical layer, mantle internal heating and lithospheric weak zones

**Figure 3.**
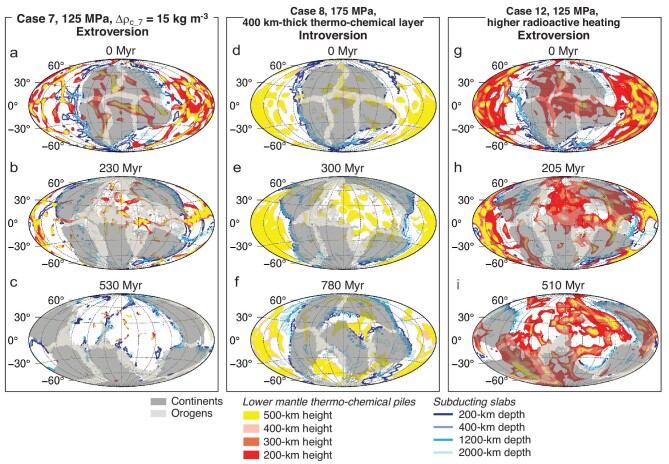
Evolutionary snapshots for Cases (a–c) 7, (d–f) 8 and (g–i) 12. Case 7 is the same as Case 1 but with a 50% reduction in density for the lower-mantle thermo-chemical layer. Case 8 is the same as Case 3 but with a thicker (400 km) initial lower-mantle thermo-chemical layer. Case 12 is the same as Case 1 except that the value for mantle internal radioactive heating is changed from that for Pangea to that for Nuna [39]. The modeling results of both Cases 7 and 12 (i.e. extroversion assembly of the daughter supercontinent) remain the same as in Case 1, and those of Case 8 remain the same as Case 3 (introversion supercontinent assembly). Note that in (d–f), only contours of the lower-mantle thermo-chemical layer at 400-km above the CMB are shown.

**Figure 4.**
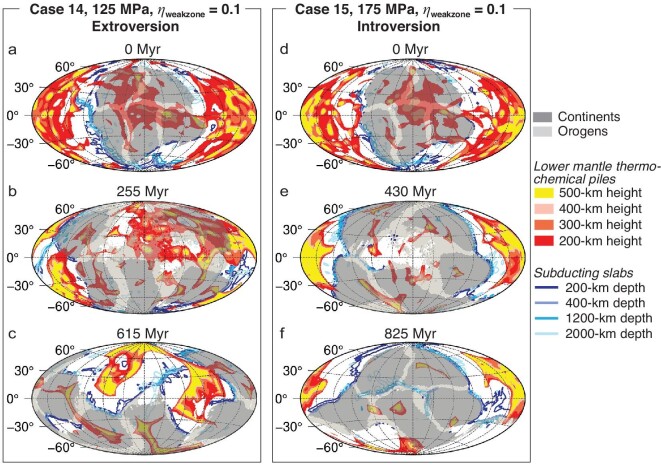
Evolutionary snapshots for Cases (a–c) 14 and (d–f) 15. Case 14 is the same as Case 1 except that the viscosity in the weak zones *η*_weakzone_ is 1/10 of the oceanic lithospheric value, 10 times higher than the default value (i.e. *η*_weakzone_ = 0.01 in Case 1). Similarly, Case 15 is the same as Case 3 but with 10 times increased viscosity in the weak zones, i.e. *η*_weakzone_ = 0.1.

We also examine the possible effects of changing the density and volume of the thermo-chemical piles/layer in the lower mantle on the supercontinent cycle. As shown in Fig. 3 and Cases 4–11 in Table S2 of Supplementary Data, whether a supercontinent is assembled through introversion or extroversion is still controlled by the strength of the oceanic lithosphere, with variations in either the density or thickness of the thermo-chemical layer at the bottom of the mantle making no difference in the model outcomes. Similarly, changing the radioactive heating rate of the mantle does not alter the model outcomes either (Fig. 3; Cases 12 and 13 in Table S2). In addition, by reducing the viscosity drop for the automatically generated weak zones along continental margins (Fig. 4; Cases 14 and 15 in Table S2), the supercontinent evolution paths remain the same as models (Cases 1 and 3) with a larger viscosity drop. It shows that the property of the weak zones used in our modeling for the generation of ocean–continent subduction does not affect the supercontinent assembly.

## DISCUSSION

### Thinning of oceanic crust with time due to Earth's secular cooling: a cause for secular changes in oceanic lithospheric strength?

**Figure 5.**
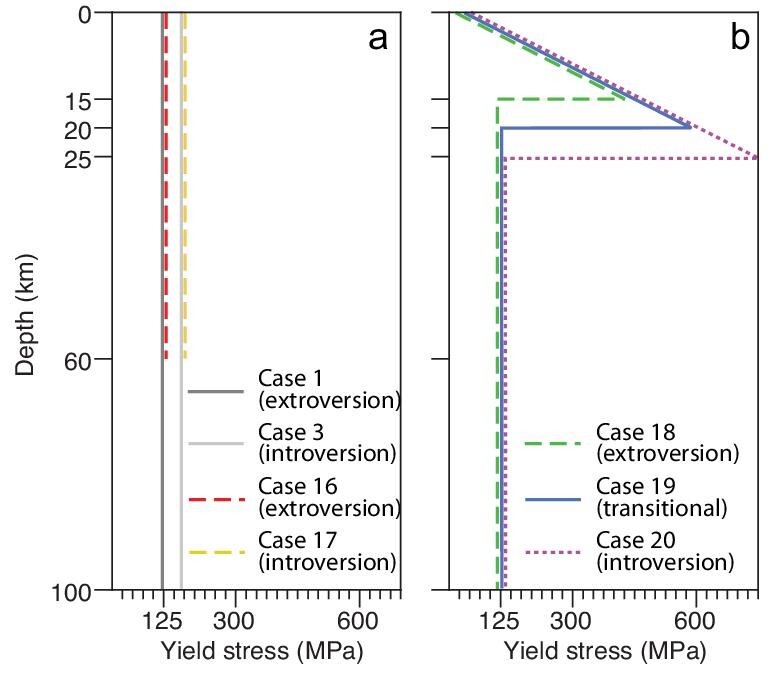
Oceanic lithospheric thickness and yield stress settings for the modeling. (a) Settings for Cases 1, 3 and 16–17, where Cases 1 and 16 both have a yield stress of 125 MPa but the thickness of oceanic lithosphere *D_olith_* changes from 100 km (Case 1) to 60 km (Case 16). Similarly, Cases 3 and 17 have the same 175 MPa yield stress, but their *D_olith_* are different. (b) Settings for Cases 18–20. In contrast to Case 1, yield stress in the oceanic crustal layer of these cases (with thicknesses set at 15, 20 and 25 km, respectively) is constrained by the linear Byerlee's law [34] (see Methods in Supplementary Data).

The strength of the oceanic lithosphere could potentially have been affected by two secular changes in Earth structure as the Earth cooled with time: a decreasing thickness of the oceanic lithosphere (Fig. 5a) [29] and/or a decreasing thickness of the oceanic crust (Fig. 5b) [30], both due to the reduced degree of partial melting as the mantle cooled (see Methods in Supplementary Data) [29–31]. We use Cases 16 and 17 to examine the effect of changing oceanic lithospheric thickness (*D_olith_*) from 100 km to 60 km (Fig. 5a) while the rest of the model set-ups remain the same as for Cases 1 and 3, respectively. As shown in Fig. S5, both cases produced supercontinent assembly in the same way as simply varying the yield strength of the oceanic lithosphere (i.e. extroversion for Cases 1 and 16, and introversion for Cases 3 and 17), rendering this factor unimportant for determining how a supercontinent is assembled. This is probably because the effective viscosity of the oceanic lithosphere beneath ∼50–60 km is dominated by the temperature- and pressure-dependent viscous branch (creep flow; see Methods in Supplementary Data), other than the brittle deformation. The former is independent of yield stress. It also implies that dehydration stiffening [32,33] due to partial melting at the lower part of the oceanic lithosphere, which potentially leads to a stronger lithospheric bottom when the mantle is hotter than the present, may not be crucial in determining the continental assembly paths.

**Figure 6.**
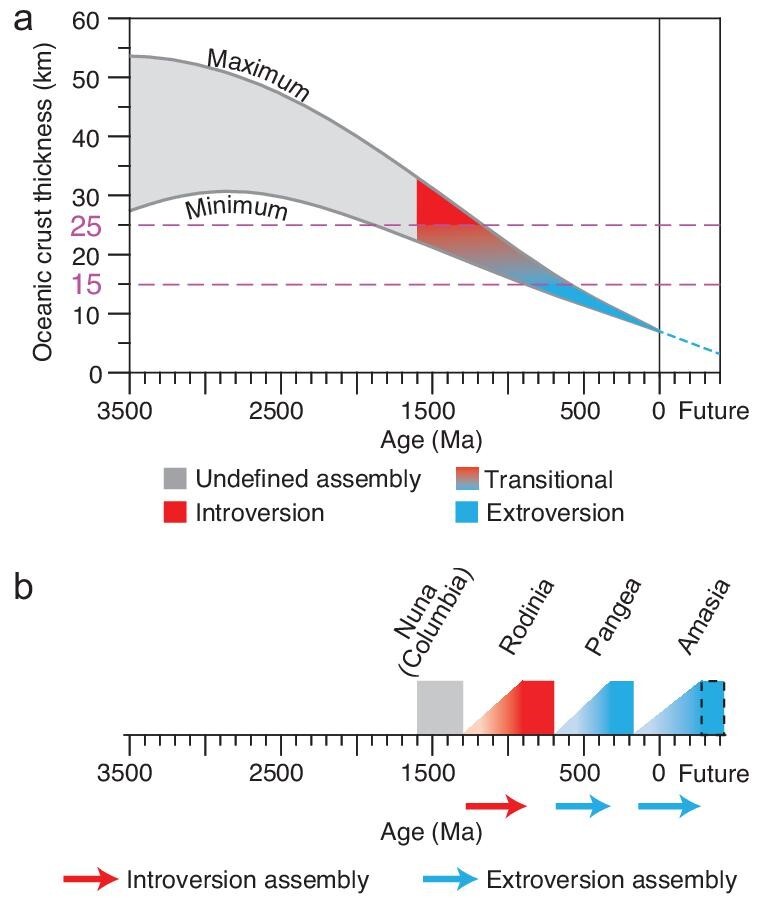
Estimated range of oceanic crustal thickness during Earth's secular cooling that determines the age ranges for the varying manners of supercontinent assembly for both geological time and the future. (a) The graph shows the relationship between Earth's age (millions of years ago) and the oceanic crustal thickness [30] based on the changing mantle potential temperature [31], and the corresponding manners of supercontinent assembly. We explored the calculated curves into the future. (b) The sketch shows the assembly of the three known supercontinents in Earth’s history and the future supercontinent Amasia [4], with model-predicted varying manner of supercontinent assembly. According to our modeling results, Amasia will assemble via the closing of the Pacific Ocean (legacy of the previous superocean) (Fig. 1c).

On the other hand, theoretical calculations predict a thicker oceanic crust in Archaean–Proterozoic time due to a higher mantle temperature [31] that led to a higher volume of basaltic melts being extracted from the mantle [30]. In Fig. 6a, we convert the estimated time evolution of the mantle potential temperature into corresponding oceanic crustal thickness [30,31], which shows a gradual thinning of the oceanic crust from ∼30–47 km at ca. 2.5 Ga to 6–7 km in the present day. We further examine the effect of changing oceanic crustal thickness on supercontinent cycle by conducting a series of cases with the thickness of oceanic crust set at 15, 20 and 25 km, respectively (Fig. 5b; Cases 18–20). In such cases, the yield strength curves in the crust (Fig. 5b) are depicted by the linear Byerlee's law [34]. The results (Fig. S6) show that supercontinent cycles are quite sensitive to the oceanic crustal thickness (*D_crust_*): when *D_crust_* is 15 km, the supercontinent assembles through extroversion; when *D_crust_* is 25 km, the supercontinent assembles through introversion; for oceanic crustal thickness between 15 and 25 km (i.e. when *D_crust_* is 20 km), the supercontinent assembles in an orthoversion fashion. These results demonstrate that how a supercontinent is assembled is primarily determined by the secular change of the global oceanic crustal thickness, with thicker crust (stronger lithosphere) leading to introversion assembly, and thinner crust (weaker lithosphere) leading to extroversion assembly. This result assumes most of the oceanic lithospheric strength resides in the crustal layer.

Another theoretical model by Korenaga [35,36] suggests that the strength of the oceanic lithosphere rests in both the crust and the lithospheric mantle until the hypothesized ‘thermal cracking’ weakens the mantle lithosphere. Exactly how the oceanic mantle lithosphere and its strength behaved through Earth's history remains to be resolved by future studies. Given the overall dominant influence of the crust over the mantle lithosphere in the strength of the oceanic lithosphere, as discussed above, such detailed knowledge is not expected to affect the first-order conclusions of our modeling results.

### A one-off occurrence of introversion supercontinent assembly in the Precambrian?

Our modeling results, in combination with Earth's cooling history (Fig. 6a), indicate that a step change from introversion to extroversion supercontinent assembly occurred between ∼1.85–1.15 Ga and ∼0.90–0.55 Ga, a period corresponding to oceanic crustal thickness of 25–15 km [30,31]. Taken together, the results suggest that if introversion supercontinent assembly ever occurred, it could only have occurred in the Precambrian time (>540 Ma). On the other hand, Phanerozoic (<540 Ma) supercontinent assembly could only occur through extroversion. Orthoversion supercontinent assembly would only be possible between these two endmember states.

The supercontinent Nuna/Columbia has widely been recognized to be Earth's first supercontinent [7,8]. Its formation may thus have involved the gradual centralization of small-scale mantle downwellings into a single super-downwelling [28] instead of introversion, extroversion or orthoversion (Fig. 1). The next supercontinent, Rodinia, is the only supercontinent that could have assembled through introversion according to our modeling results (Fig. 6b), and the Phanerozoic supercontinent Pangaea could only have been assembled through extroversion. Such results are consistent with the model of Hoffman [37] and Li and Zhong [4] based on paleogeographic reconstructions.

### The assembly of the future supercontinent Amasia through extroversion?

Our results preclude the possibility of the future supercontinent Amasia [14] being assembled through either introversion, by closing the Atlantic and Indian oceans (Fig. 1e) [11], or orthoversion, by closing Arctic and Caribbean seas (Fig. 1d) [13]. Instead, Amasia could only have an extroversion assembly through the closure of the Pacific Ocean (Fig. 1c) [4,37] due to the weakening of the oceanic lithosphere with time.

### Lifespan of LLSVPs

Our modeling results also have predictions on the dynamic evolution of LLSVPs (or mantle superplumes) during the supercontinent cycle. According to our models, the shapes and distribution of the thermo-chemical piles in the lower mantle are primarily driven by subduction geometry [38]. The LLSVP that formed beneath the mother supercontinent can survive over two supercontinent cycles if the daughter supercontinent is assembled through extroversion (Movie S1) [11], but in such a case the LLSVP under the original superocean-1 gets destroyed by the assembly of the daughter supercontinent. The LLSVP under the original external superocean-1 can only survive beyond one supercontinent cycle if the daughter supercontinent is assembled through introversion (Movie S3) [11,38]. Our model therefore predicts the destruction of the present Pacific LLSVP by the future extroversion assembly of Amasia.

### Possible effect of diffusive mid-ocean ridges in the modeling

Spreading ridges feature in our models as being diffusive (e.g. viscosity field in Movie S1 of Supplementary Data) instead of being divergent linear features. Linear spreading ridges were used in models with strongly temperature-sensitive mantle viscosity (changing by >1e6 times when the non-dimensional temperature changes from 0 to 1), where chemically distinct continents are absent [27]. In comparison, we used temperature-induced viscosity changes of ∼1e4 times in this work. The resulting diffusive ridges in our models produce smooth velocity fields, but have little effect on the age of the oceanic lithosphere, especially near the edges of the oceans, thus also negligible influence on the ocean–continent subduction induced by weak zones and ultimately the modeling results.

## CONCLUSIONS

To summarize, our modeling work suggests that the strength of the oceanic lithosphere, primarily controlled by the thickness of the global oceanic crust, determines how a supercontinent is assembled. Earth's secular cooling since the Archean time has led to a gradual thinning of the oceanic crust with time, meaning that introversion supercontinent assembly could only have occurred in the Precambrian, whereas for the Phanerozoic and into the future, supercontinents could only be assembled through extroversion, i.e. the closure of the external superoceans. This predicts that the next supercontinent, Amasia, could only be assembled through the closure of the Pacific Ocean.

## DATA AVAILABILITY

All the data and resources that are necessary for evaluating or reproducing the findings of this study (including source code and data for figures) are available at https://data.mendeley.com/drafts/bbwfs9p2nk.

## Supplementary Material

nwac205_Supplemental_Files
