# Supplementary material for: Will Earth's next supercontinent assemble through the closure of the Pacific Ocean?
Source: Natl Sci Rev. 2022 Sep 28;9(12):nwac205. doi: 10.1093/nsr/nwac205 (PMC9743166; doi:10.1093/nsr/nwac205)
Supplement: nwac205_Supplemental_Files [file nwac205_supplemental_files.zip › NSR_20220805_suppPDFin_author corrected with Proof-new.docx]

Supplementary Data for

Will Earth’s next supercontinent assemble through the closure of the Pacific Ocean?

**Authors:** Chuan Huang^1,2*§^, Zheng-Xiang Li^1*§^, Nan Zhang^2,1^

**Affiliations:**

^1^Earth Dynamics Research Group, The Institute for Geoscience Research (TIGeR), School of Earth and Planetary Sciences, Curtin University, Perth, GPO Box U1987, WA 6845, Australia

^2^Key Laboratory of Orogenic Belts and Crustal Evolution, School of Earth and Space Sciences, Peking University, Beijing 100871, China

*Corresponding author. Email: chuan.huang@curtin.edu.au; [Z.Li@curtin.edu.au](mailto:Z.Li@curtin.edu.au)

^§^These authors contributed equally to this work.

**Contents of this file**

Methods

Figures S1 to S9

Tables S1 to S2

Captions for Movies S1 to S3

References for Supplementary Data

**Methods**

*Governing equations:*

We build self-consistent dynamic models in a 3-D spherical domain with Extended Boussinesq Approximation. The non-dimensional conservation equations for mass, momentum, energy, and chemical compositions are [1]:

$\nabla\cdot\text{u}=0$ (1)

$-\nabla p+\nabla\cdot\left[ \eta_{e}\left( \nabla\text{u}+\nabla\text{u}^{T} \right) \right]-\left( RaT\alpha-\sum_{i} {Ra}_{c\_i}f_{i}(C_{i}) \right)\hat{\text{g}}=0$ (2)

$\frac{DT}{Dt}-Di\alpha\hat{\text{g}}\text{u}\left( T+T_{s} \right)=\nabla^{2}T+H+\Phi$ (3)

$\frac{\partial C_{i}}{\partial t}+\text{u}\cdot\nabla C_{i}=0$ ， (4)

where **u**, *p*, *η_e_,* *T*, $\hat{\text{g}}$, *T_s_*, *H*, Φ are the velocity vector, dynamic pressure, effective viscosity, temperature perturbation, unit gravity vector, surface temperature, internal heating rate, and viscous heating, respectively. *f_i_* is the fraction of the *i*th chemical composition *C_i_*, and *Di* is the dissipation number. Rayleigh number *Ra* and chemical Rayleigh number *Ra_c_i_* are defined as

$Ra=\frac{\alpha_{0}\rho\text{g}\Delta TR^{3}}{\kappa\eta_{ref}}$ (5)

${Ra}_{c\_i}=Ra\frac{{\Delta\rho}_{c\_i}}{\alpha_{0}\rho\Delta T}$. (6)

Here, the variables are dimensional surface thermal expansivity *α*_0_, density *ρ*, gravity *g*, temperature contrast across surface and the core-mantle boundary *ΔT*, Earth’s radius *R*, thermal diffusivity *κ*, reference viscosity *η*_ref_, and chemical extra density *Δρ_c_i_* (Table S1). Thermal expansivity *α* in Eq. (2–3) changes with depth *z* (normalized by radius *R*) and is written as [2]

$\alpha(z)=\alpha_{0}/{(1+mz)}^{3}$, (7)

where *m* = 1.5778, which gives 5-times decreased thermal expansivity from surface to the CMB, and matches the experimental data [3]. Since the strong spatially variable *α* is also included in the dissipation number *Di*, we choose the averaged *Di* over depth as its representative value, i.e., *Di_effective_* as in Čížková et al. [4] (Table S1).

Rheology law in our model contains two branches. One depicts the creep flow where the rheology (defined by viscosity$\eta_{t}$) is depth-, temperature-, and composition-dependent and is expressed by formula:

$\eta_{t}=\eta_{r}\left( z \right)(\prod_{i} \eta_{c}\left( C_{i} \right)^{f_{i}})exp(\frac{E}{T+T_{o}}-\frac{E}{T_{r}+T_{o}})$, (8)

where *η_r_*(*z*) and *η_c_*(*C_i_*) are pre-factors due to changes in depth and composition, respectively, *E* is the activation energy, *T_r_* the mantle reference temperature, and *T_o_* the temperature offset (Table S1). The other branch parameterizes the brittle and plastic (the time-independent component) deformation [5] as:

$\eta_{y}={\sigma_{y}(C_{i})}/{2\dot{\varepsilon}_{II}}$, (9)

where *σ_y_*(*C_i_*) and $\dot{\varepsilon}_{II}$ are the yield stress and the second invariant of strain-rate tensor in the lithosphere, respectively (Table S1). The final effective viscosity *η_e_* in Eq. (2) is calculated through equation:

$\eta_{e}=\left( 1/{\eta_{t}}+1/{\eta_{y}} \right)^{-1}$. (10)

The reference viscosity of our model is 8×10^21^ Pa·s, chosen at the viscosity right below the 660km-discontinuity, corresponding to a Rayleigh number of 1×10^8^. The calculation domain contains 64×64×64×12 elements with refinements in the upper 40 km (~5 km per element) and lower 150 km (~25 km per element). In all the calculations, free-slip and isothermal conditions apply to the top and bottom boundaries. The modelled supercontinent consists of five continental plates covering ~30% of Earth’s surface area. Each continent is tracked by its individual tracers, as for the orogens and the thermo-chemical layer at the bottom of the mantle. Our models therefore involve seven chemical compositions (i.e., index *i* in Eq. (2) ranges in $1,\cdots, 7$).

*Initial conditions:*

To obtain the initial temperature condition, we first run a pure thermal convection model to generate a degree-1 temperature structure featuring a large mantle super-upwelling and an antipodal super-downwelling (Figure S7a) [6] that typically accompany supercontinent assembly [6,7]. We then place a supercontinent consisting of five continents over the super-downwelling to imitate the assembly of a supercontinent, and plant a lower mantle chemical layer above the CMB (Figure S7b). Self-generated circum-supercontinent subduction (the subduction girdle) then leads to the formation of two broad, antipodal hot anomalies in the lower mantle, representing the LLSVPs (Figure S7c). The enduring plume activities over the sub-supercontinent LLSVP [8], along with subduction retreat, then facilitate supercontinent break-up [9,10]. Continents start to drift independently with supercontinent break-up, and we choose this time as the starting point of examining how the moving continents and mantle dynamics evolve into the assembly of the next supercontinent. Note that the circum-supercontinent subduction will evolve into a fully-closed “subduction wall” if the preparation stage goes beyond ~200 Myr [10], which would result in the subduction retreat and supercontinent breakup becoming unfeasible [10].

*Yield stress in oceanic lithosphere:*

Uniform yield stresses of 400 MPa [11] and 50 MPa [12] are applied to cratons and orogens, respectively, in this work. In contrast, a simplified layered strength profile is applied to the oceanic lithosphere. Laboratory experiments suggest three distinct rock deformation regions in the oceanic lithosphere [13,14]: 1) a brittle crustal layer, 2) plastic flow (including low-temperature plasticity and creeping) for the lower lithosphere where the temperature is above ~400 °C, and 3) a semi-brittle layer between (1) and (2) (Figure S8). Oceanic lithospheric strength in the semi-brittle region has not been well determined, and the strength in the plastic flow region is low and follows the flow law [15]. Therefore, as shown in Figure S8, the strength of the oceanic lithosphere concentrates mainly in two parts: the lower crustal layer (may extend into the semi-brittle region with a thick oceanic crust) and the region atop of the low-temperature plasticity region. Furthermore, numerical calculations by fitting the topography of ocean islands suggest that the strength in the plastic region (100–200 MPa) [14] is significantly lower than the laboratory-derived values (up to ~800 MPa) [15]. The former is also consistent with the values (~100–200 MPa) suggested by a wide range of mantle convection modeling work (e.g., [5,16–18]). The discrepancy between modeling and laboratory experiments-derived oceanic lithospheric strengths may be due to the extremely higher strain rates used in laboratory experiments which are ~10 orders of magnitude higher than those in nature (10^–6^–10^–4^ s^–1^ vs. 10^–17^–10^–14^ s^–1^), which poses questions regarding the reliability of the experimental data [19]. This work generally follows the modeling-suggested oceanic lithospheric strength (orange region in Figure S8) and simplified the oceanic lithosphere profile to one-layered or two-layered models as below.

For the one-layered model, constant oceanic lithospheric yield stresses (Cases 1–17 with values varying between 125, 150, and 175 MPa) are used to estimate the overall effect of plastic yielding in the oceanic lithosphere on the model results. This simplification is useful for examining the overall effects of oceanic lithospheric strength on the evolution of supercontinent assembly. For the two-layered model, we use a linearly increasing yield stress profile for the crustal layer [20] expressed as $\sigma_{y}\left( C_{0} \right)=\sigma_{0}+\mu_{f}\sigma_{n}$, where $\sigma_{0}$,$\mu_{f}$, $\sigma_{n}$ are the frictional cohesion, frictional coefficient, and hydrostatic pressure, respectively. We choose $\sigma_{0}=20 \text{MPa}$ [21] and $\mu_{f}=0.85$ [20], and keep the strength for the mantle lithosphere constant. The two-layered model assumes that the strength of oceanic lithosphere mainly resides in its crustal layer (Cases 18–20; see the main text).

The significance of a given yield stress diminishes when temperatures become higher than ~800°C (over 50–60 km depth) (Figure S7d) because the deformation of the lithosphere here is controlled by diffusion and dislocation creep where the creep branch of the rheology law (which is temperature and pressures dependent; Eq. (8)) takes over the effective viscosity of the oceanic lithosphere [14]. Since the strength of the oceanic lithosphere here is irrelevant to its effective viscosity, the models’ outcomes do not alter (Cases 16–17; Figure S5; also see the main text).

*Ocean-continent subduction:*

Plastic yielding in oceanic lithosphere allows ocean-ocean subduction to occur in numerical simulations [18,22]. However, self-consistent ocean-continent subduction has seldomly been seriously considered in complex dynamic modeling with Earth-like parameters. One feasible way to generate subduction along ocean-continent boundaries is to implement a weak zone on the ocean side of such a boundary [23], realized by assigning such a weak zone a new composition with lower viscosity [24]. However, such an approach suffers from quick mantle remixing of such weak zones into the mantle due to their high fluidity, making it unsuitable for long-term (~700 Myr) Earth dynamics modeling such as ours.

In this study, special tracers are assigned on the continent-side along ocean-continent boundaries, used for tracking the weak zones, so that they do not get lost into the mantle due to oceanic subduction. These unique markers move with continents but have no density, thus no impact on the dynamic process. The tracers are initialized at 200 km away (*d_edge_*) from the continental edge when the adjacent oceanic lithosphere is older than 200 Myr (the beginning time for self-induced subduction due to gravitational instability). They are removed when the age of the adjacent oceanic lithosphere becomes less than 10 Myr (for example, when a spreading ridge approaches; Figure S9). The age of oceanic lithosphere is obtained from surface heat flow with:

$t_{ocean}=\frac{k^{2}{{\Delta T}_{lith}}^{2}}{\pi\kappa q^{2}}$, (11)

where *t_ocean_*, *k*, *ΔT_lith_*, and *q* are the age of oceanic plate, thermal conductivity, temperature of the asthenosphere, and surface heat flux, respectively (Table S1). The width of the weak zones is set at 200 km. At every execution step of the program, after the update of the special tracking tracers, the minimum distance *d_min_* between each ocean element and the gathered tracking tracers from all computation cores will be determined. Elements with *d_edge_* < *d_min_* < 400 km (200-km width weak zone + 200-km *d_edge_*) are considered as being located in the weak zones (Figure S9) and will have the viscosity *η_weakzone_* decreased to 0.01 or 0.1 times of their original values.

*Automatic generation of orogens:*

Orogens form due to the collision of continents. Orogens (also known as mobile belts) typically have thinner and weaker lithosphere than continental cratons, and can thus provide guidance for the later break-up of assembled continents [25].

To automatically generate orogens when two continents are about to collide, the program monitors the minimum oceanic span between approaching neighboring continents. If the distance becomes less than 400 km (the width of orogens used in this work), the program turns this oceanic region into a newly formed orogen with changed properties (including decreased density and yield stress; Table S1).


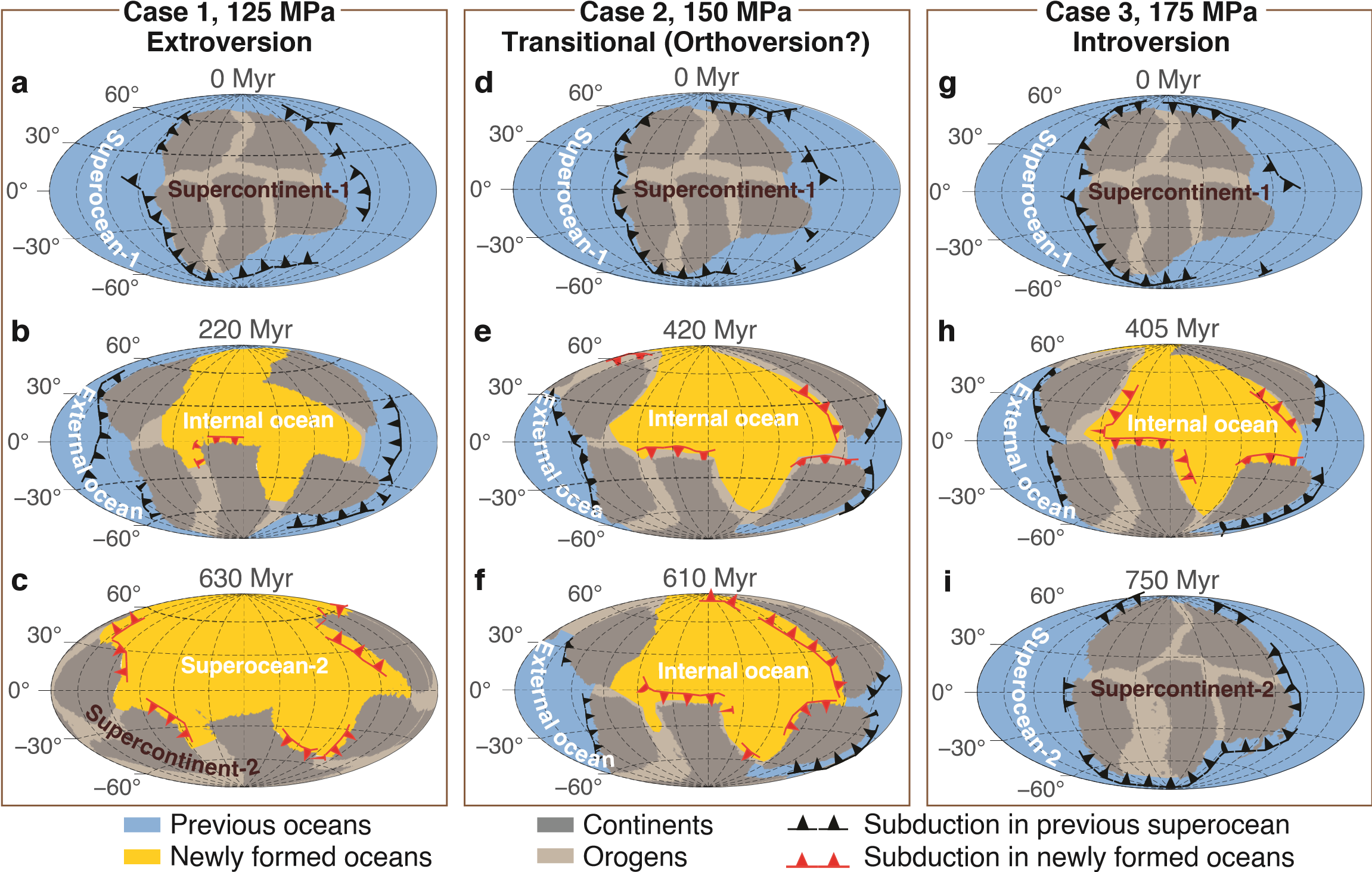


**Figure S1.** Critical paleogeographic stages of three possible ways of supercontinent assembly from the same initial paleogeographic configuration based on our modeling results. **a** to **c**, Model Case 1, extroversion supercontinent assembly with oceanic lithospheric strength of 125 MPa, where the previous superocean-1 (shown in blue) gets consumed and the new internal oceans (yellow region) grows into the new superocean-2. The old subduction girdle surrounding superocean-1 (shown with black barbed lines) and the newly formed subduction in the expanding internal ocean (shown with red barbed lines) are also shown. The dark gray regions show the positions of continents, and the areas of light gray are orogens. **d**-**f** and **g**-**i**, Same as with **a**-**c**, except that **d**-**f** (Case 2) show transitional (orthoversion?) supercontinent assembly with oceanic lithospheric strength being 150 MPa, whereas **g**-**i** (Case 3) show introversion supercontinent assembly with oceanic lithospheric strength being 175 MPa.


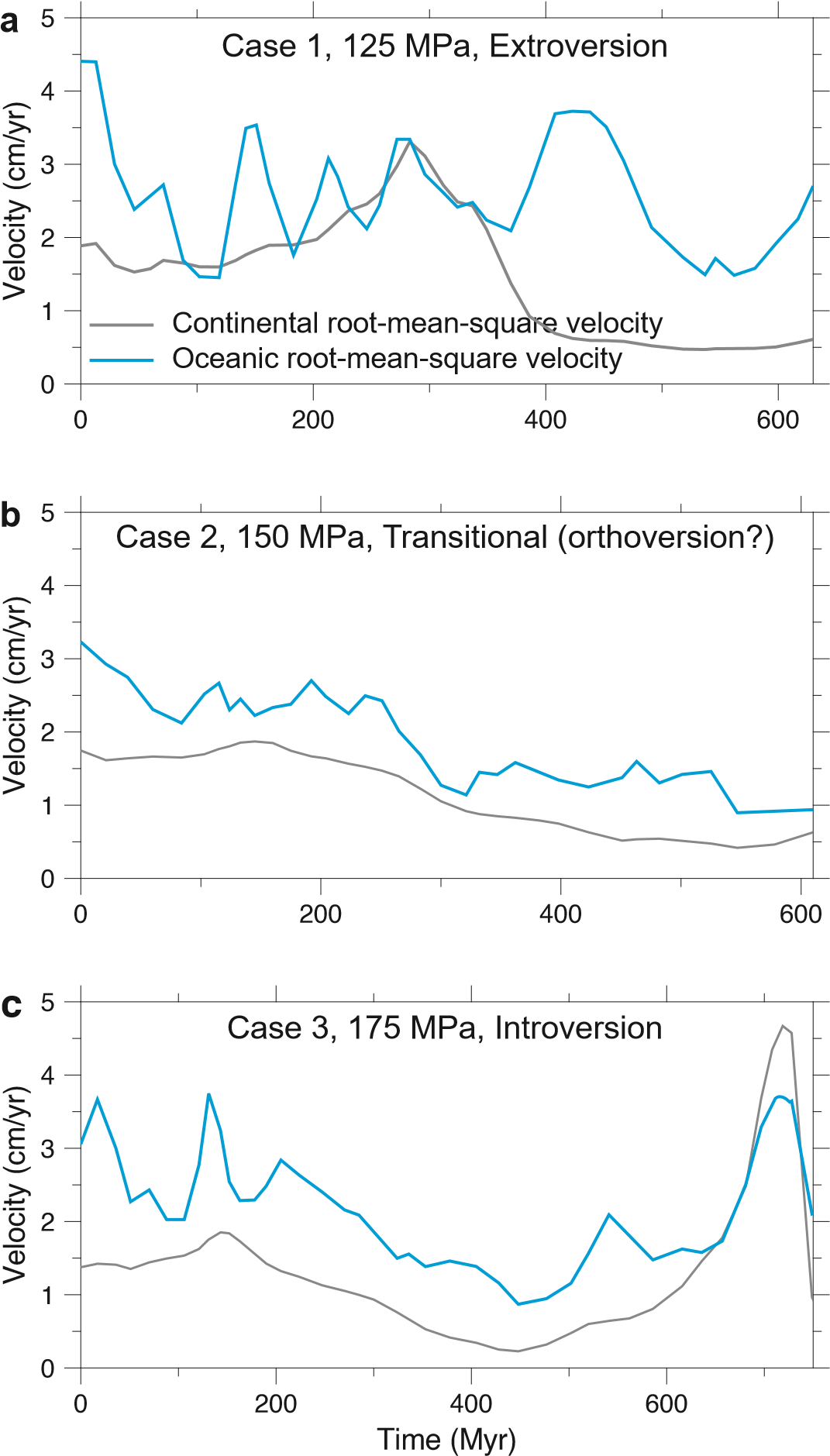


**Figure S2.** Variations of root-mean-square velocities of the continental blocks (gray line) and purely oceanic plates (blue line) with time for Cases 1–3 (**a** to **c**, respectively).


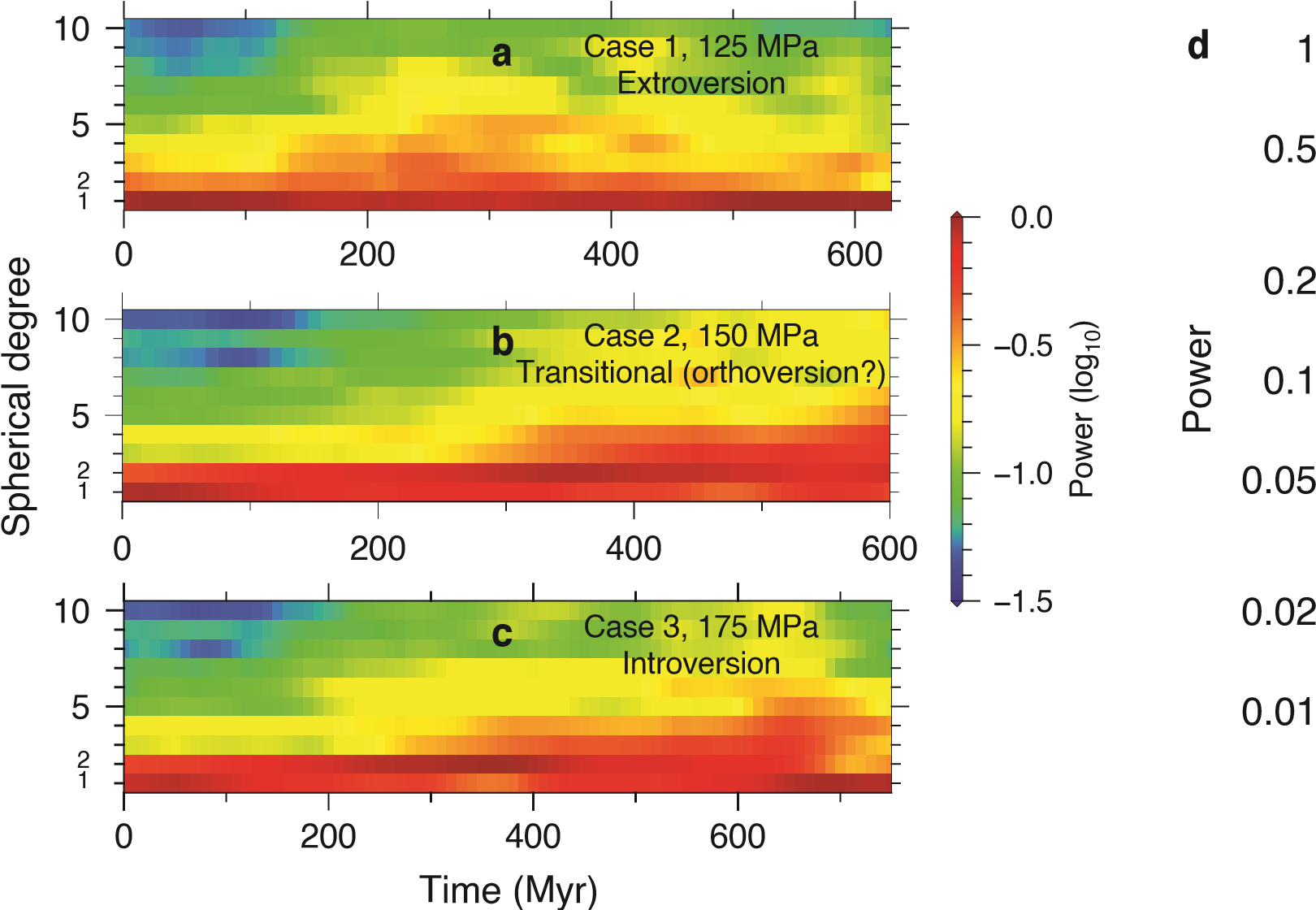

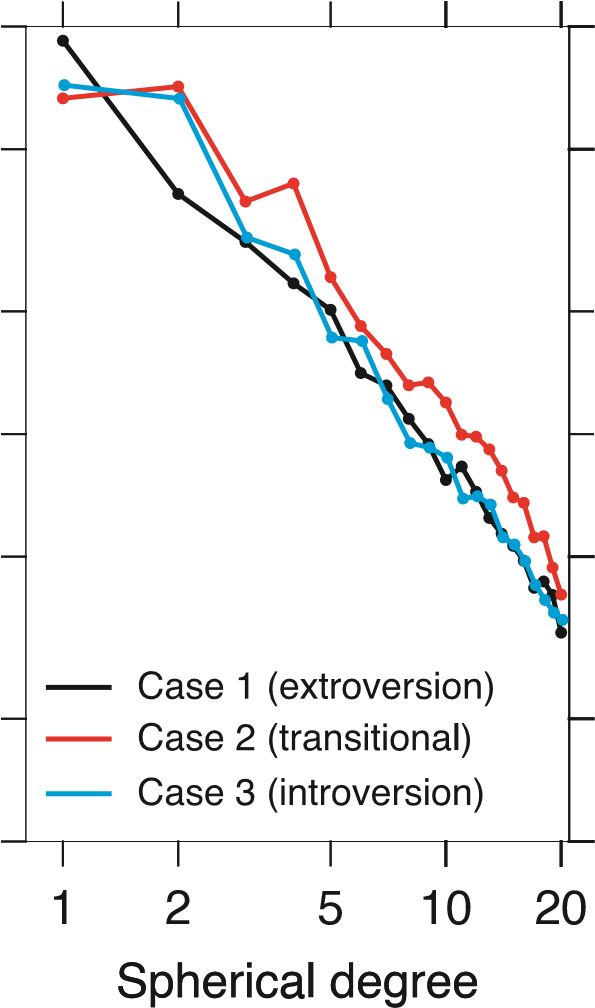


Figure S3. Spherical power of mantle structures during three different ways of supercontinent assembly. a-c, Time-dependent spherical power (including degree-1 to degree-10) for Cases 1–3. d, The average spherical power over the whole evolution time for each case.


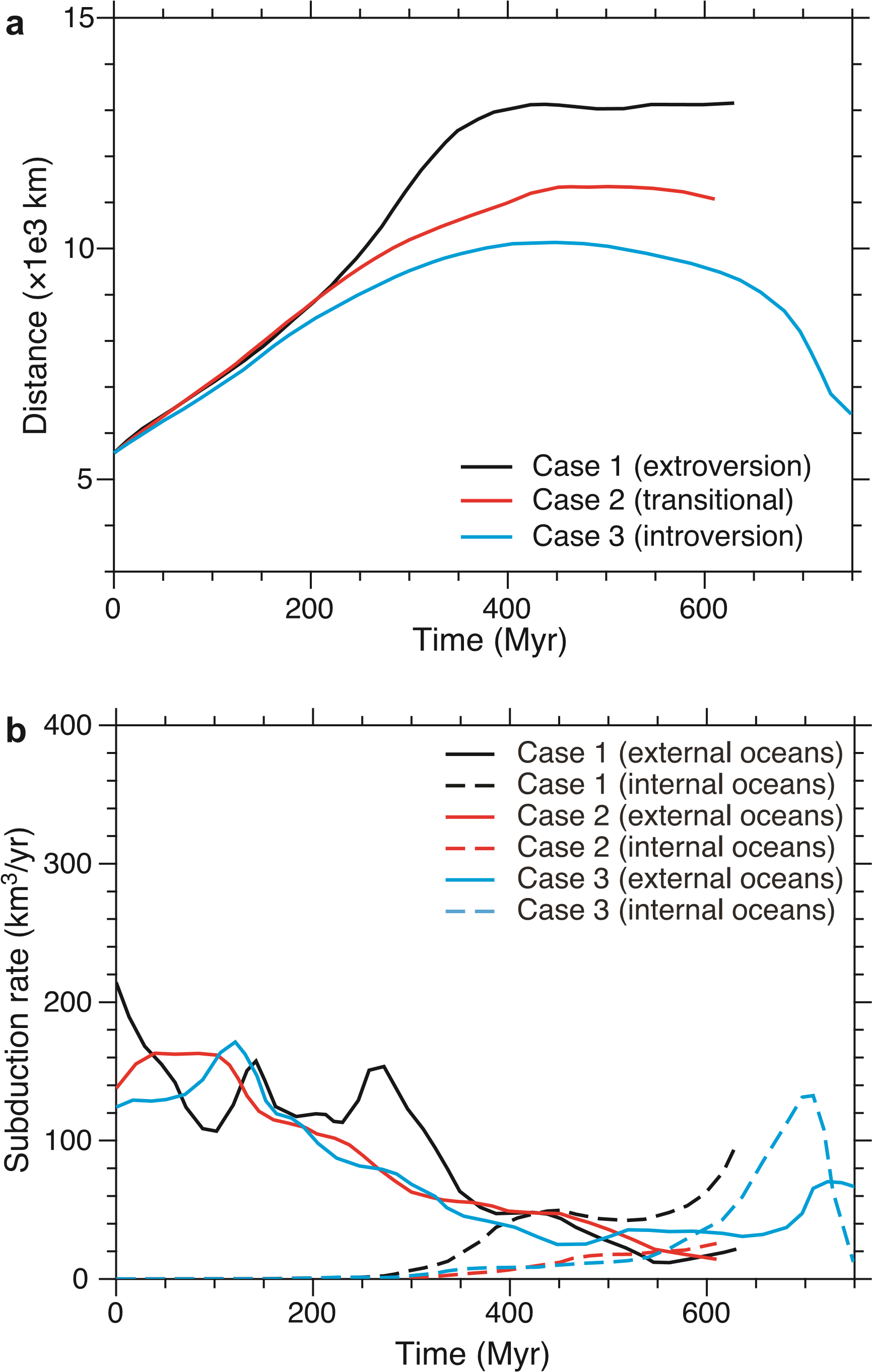


Figure S4. Varying degrees of continental scattering and subduction rates for Cases 1–3. a, Time-dependent average distance of the continental blocks from the initial supercontinent centre (longitude = 0°, latitude = 0°) for Cases 1–3, respectively. b, The time evolution of oceanic subduction rates for the external (solid lines) and internal (dashed lines) oceans of the three test cases. The subduction rate is calculated by integrating *L_e_*·*v_e_*·*D_olith_* for every subducting segment in the external (or internal) oceans, where *L_e_* is the length of the segment, *v_e_* is subduction velocity, and *D_olith_* is the thickness of oceanic lithosphere.


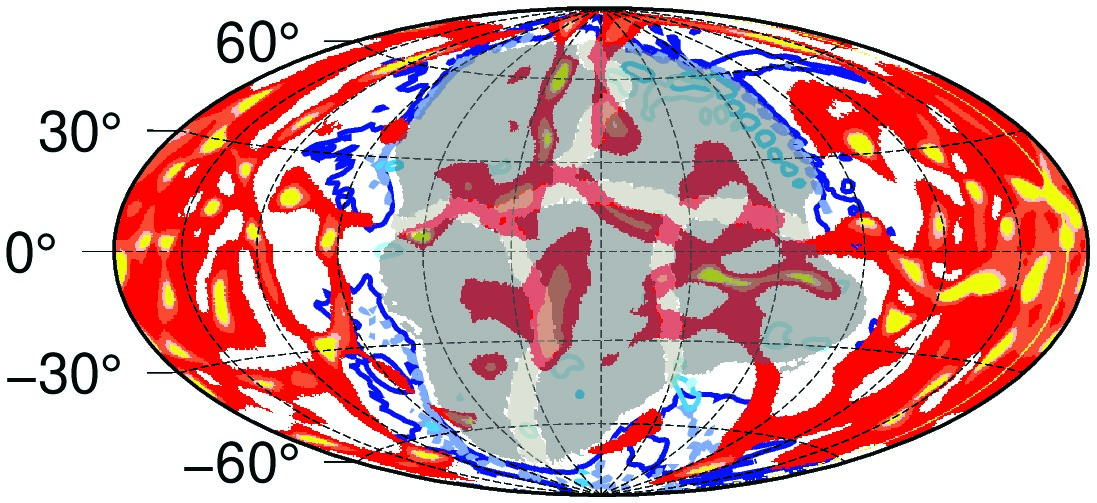

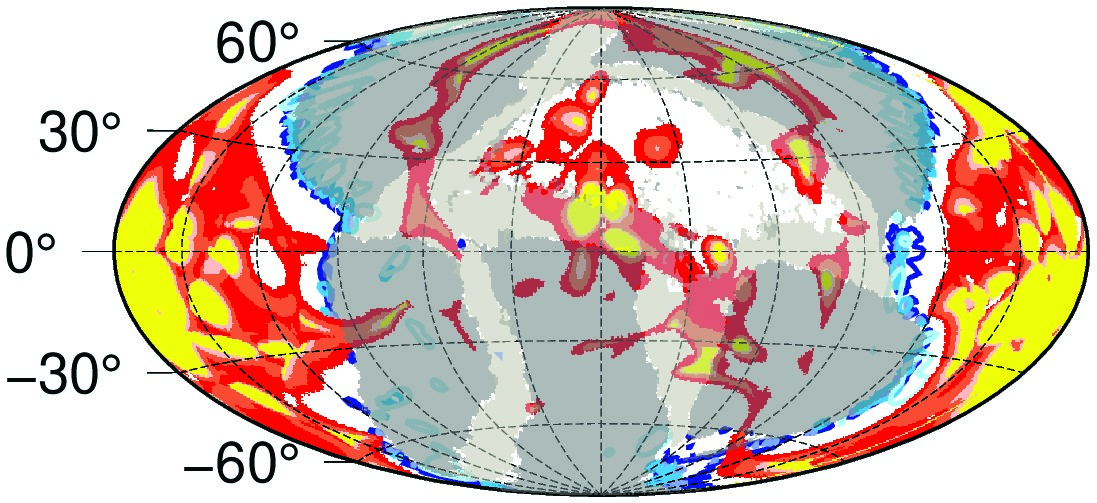

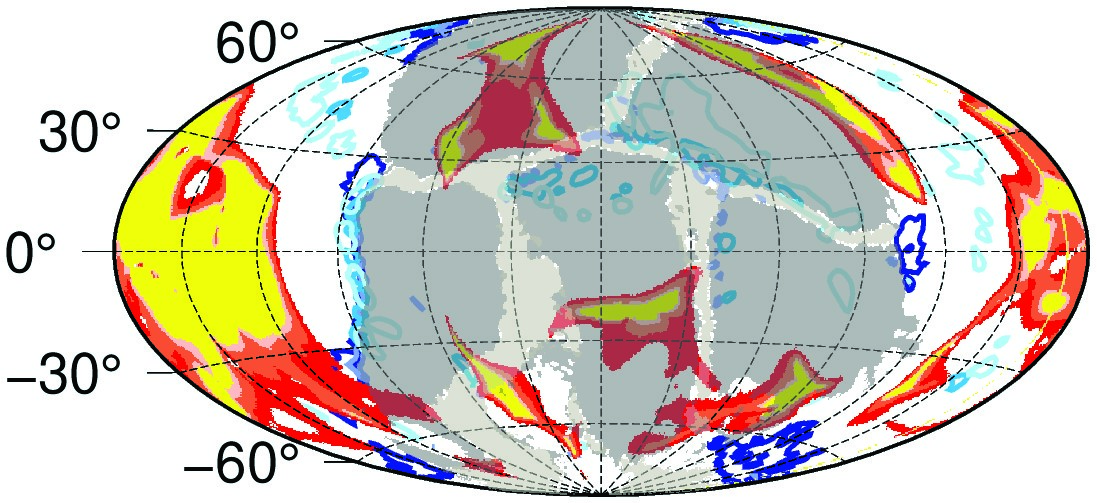

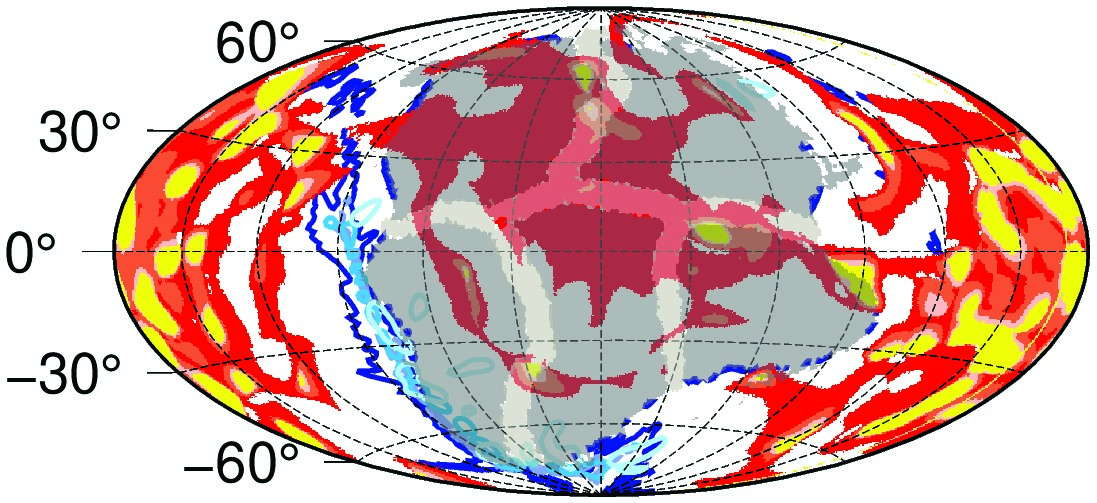

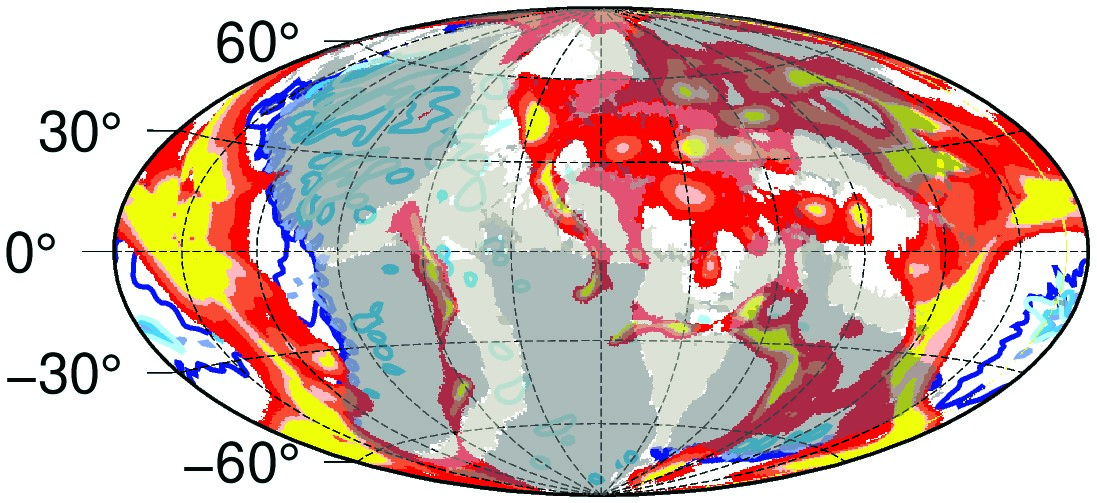

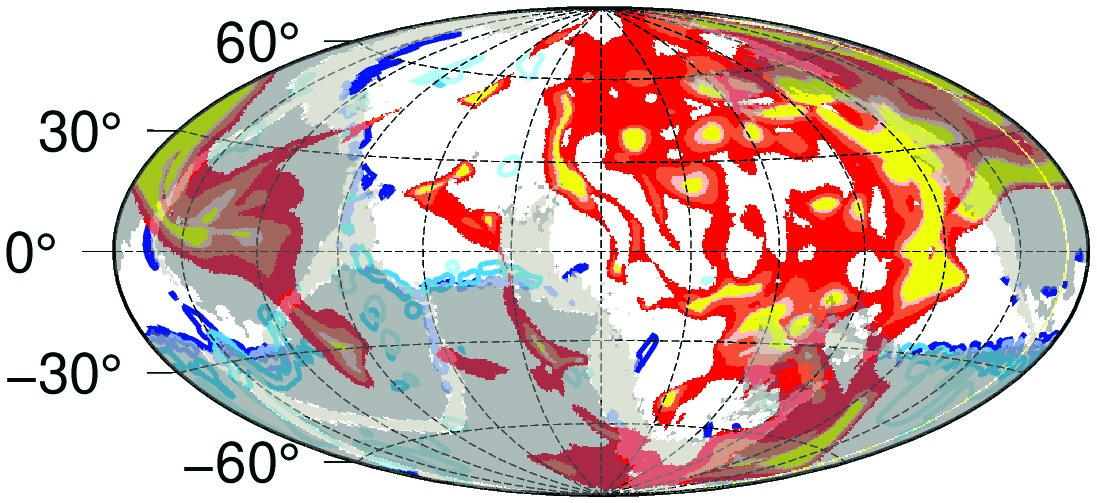


**Case 16, 125 MPa,**

***D***

**olith**

**= 60 km**

**Extroversion**

**a**

**c**

**b**

**d**

**f**

**e**

**Case 17, 175 MPa,**

***D***

**olith**

**= 60 km**

**Introversion**

0

Myr

Myr

225

590

Myr

Myr

0

Myr

430

750

Myr

500-

km height

400-

km height

300-

km height

200-

km height

*Subducting slabs*

*Lower mantle thermo-*

*chemical piles*

Continents

Orogens

200-

km depth

400-

km depth

1200-

km depth

2000-

km depth

Figure S5. Evolutionary snapshots for Cases 16 (a-c) and 17 (d-f). They are otherwise the same as Cases 1 and 3, respectively, except that the oceanic lithospheric thickness is set at 60 km here. The dark and light gray regions are continents and orogens, respectively. Topography of the lower mantle thermo-chemical layer over 200-km above CMB are shown by red-to-yellow color gradients, whereas subducted cold slabs are drawn by colors of deep blue to blue.


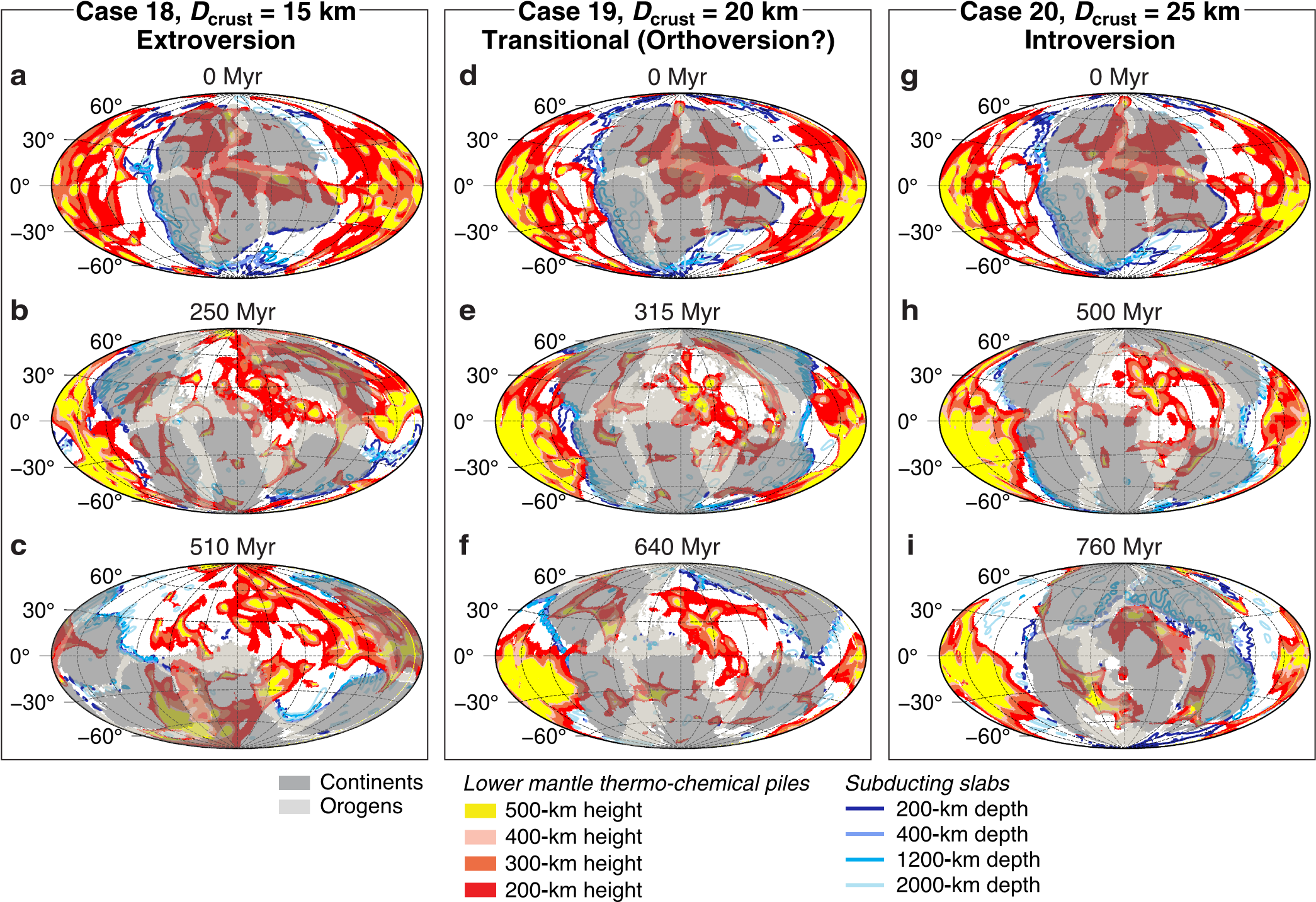


Figure S6. Evolutionary snapshots for Cases 18 (a-c), 19 (d-f) and 20 (g-i). The yield stress for the oceanic lithosphere in all three cases are set at 125 MPa, but each case has a varying thickness of oceanic crust at 15, 20 and 25 km, respectively.


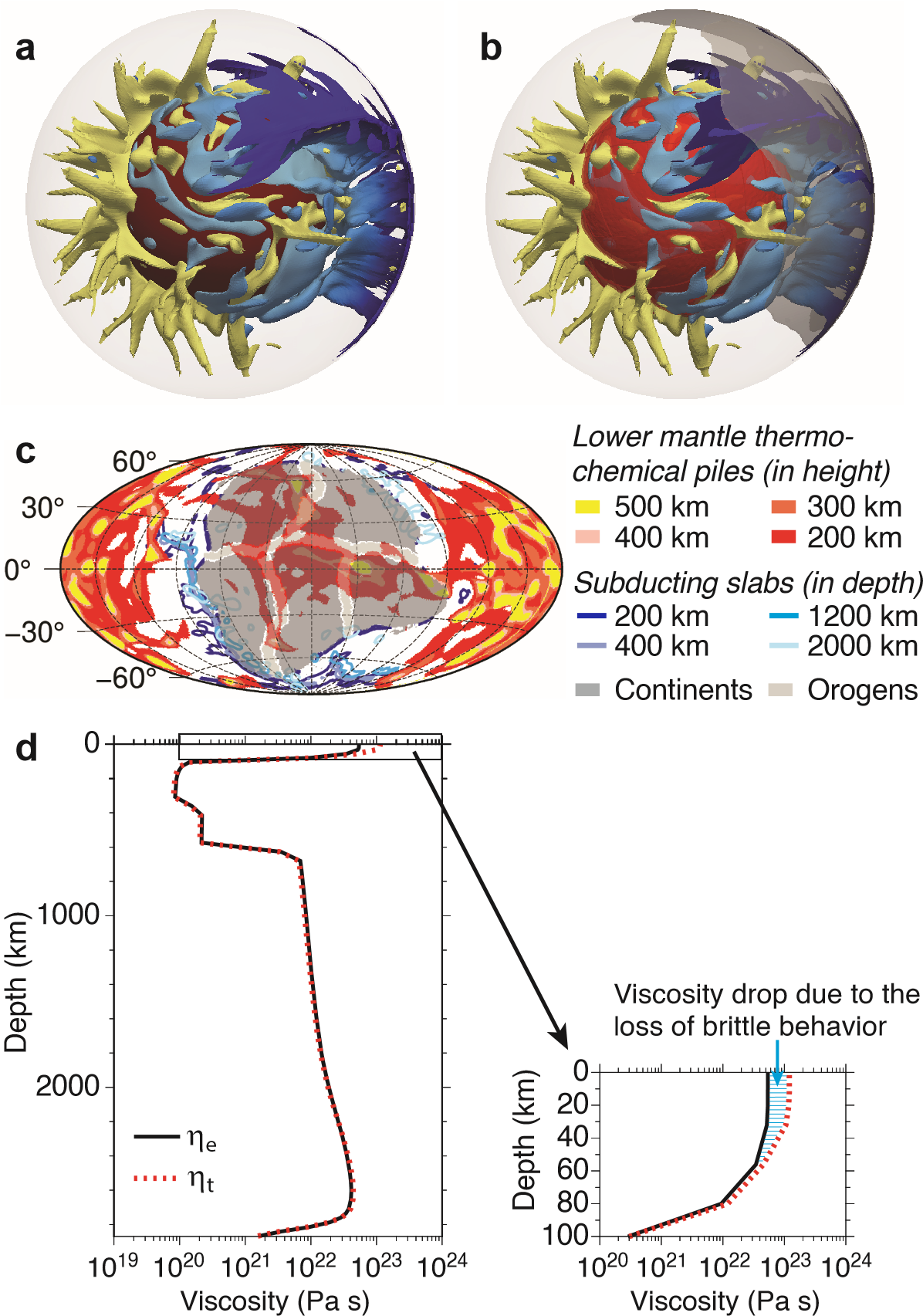
Figure S7. Model setup. a, Degree-1 mantle structure prepared in a pure thermal convection model, using the same parameters as for Case 1 (Tables S1 and S2), with upwellings shown in yellow and downwellings shown in blue. b, A supercontinent consisting of five continental blocks (semi-transparent gray regions) is then planted over the super-downwelling, and a lower mantle thermo-chemical layer (red region) planted over the CMB. c, Thermal state at the beginning of Case 1 is obtained by running mantle convection based on b for ~200 Myr until the formation of two antipodal LLSVPs (shown in red-to-yellow zones). The yellow and blue regions in a and b represent the 0.1 and −0.1 non-dimensional residual temperature iso-surfaces, respectively. The CMB is colored in dark red in a. c also shows the positions of cold slabs at various depths (with deep blue to blue contours). d, The initial viscosity profiles (including *η_e_* and *η_t_* as mentioned in Methods) of the mantle and the oceanic lithosphere for Case 1, with a zoom-in view (right panel) showing the viscosity drop (thin blue lines) in the oceanic lithosphere due to the diminishing strength with depth.


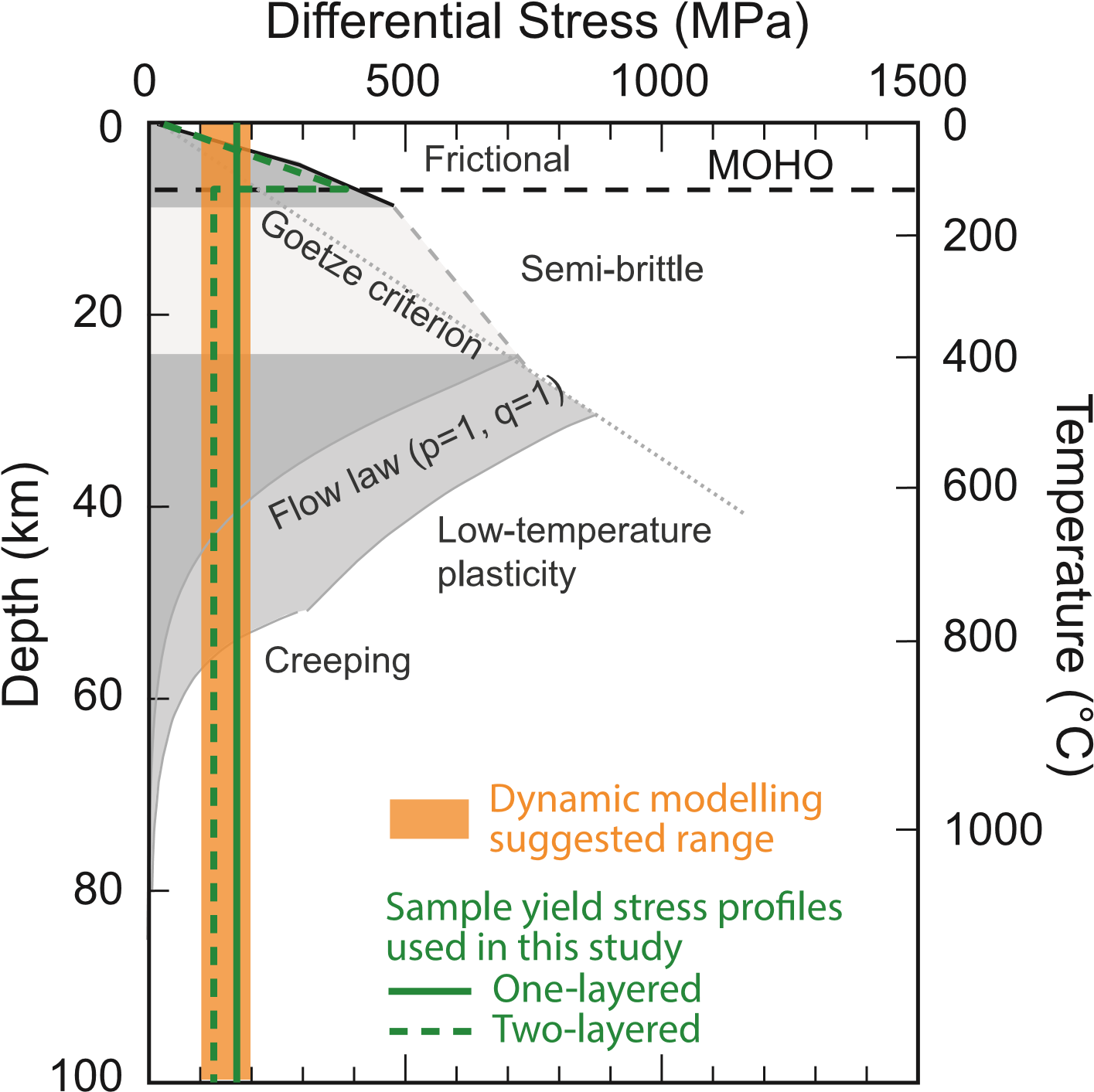


**Figure S8.** A Sketch of strength profiles for the oceanic lithosphere based on laboratory (shown in gray) and numerical experiments (shown in orange; modified from [14]). The laboratory-defined three distinct lithospheric deformation regimes (in gray) are frictional sliding, semi-brittle, and plastic flow. The curve of flow law gives the lower boundary of the yield stress in the plastic flow region. The solid and dashed green lines are the one-layered and two-layered yield stress sample profiles, respectively, that are used in this work.


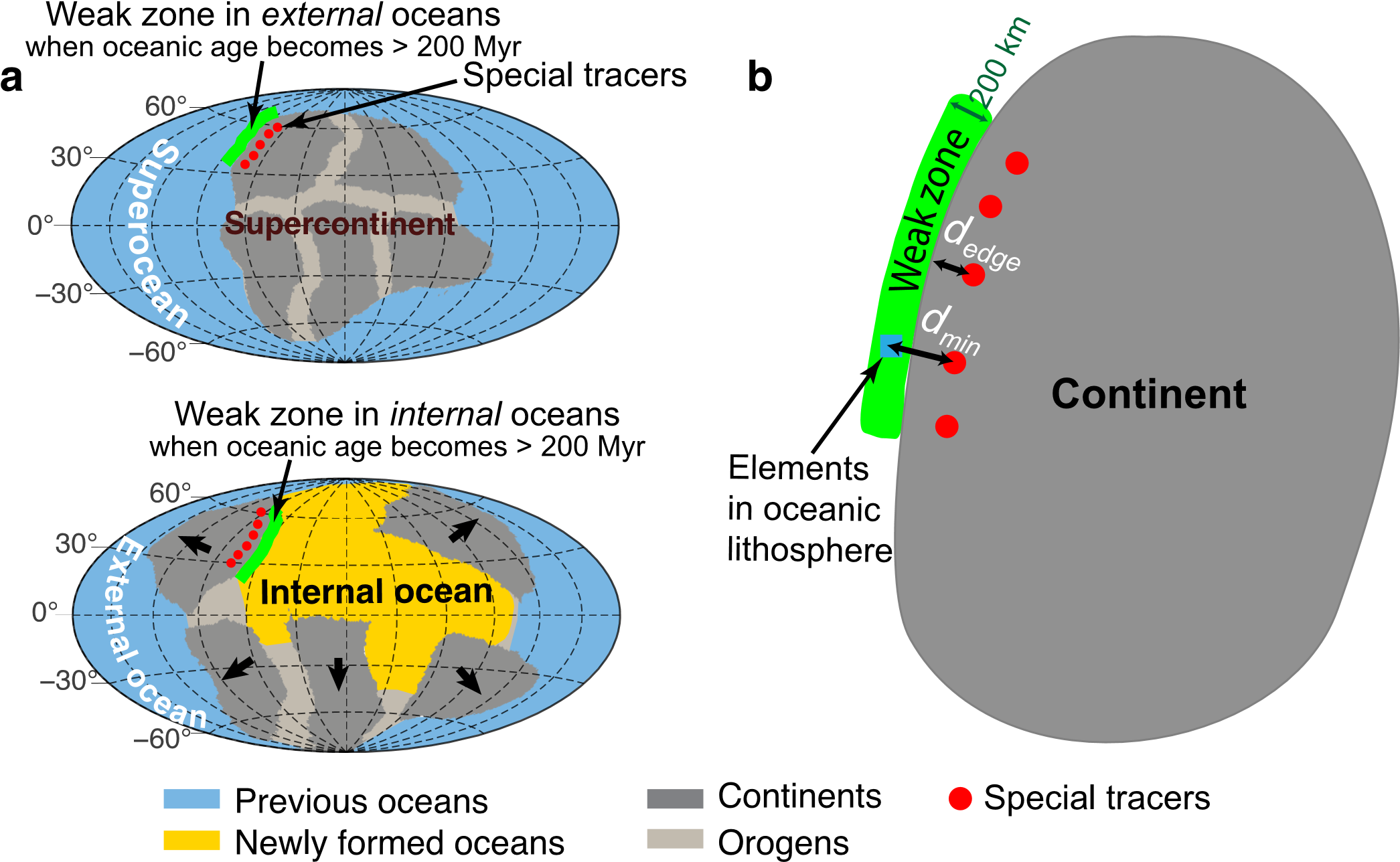


Figure S9. Sketches illustrating the automatically generated weak zones. a, Weak zones are marked by special tracers along the adjacent continental margin which are assigned when the oceanic age near the continental margin is greater than 200 Myr and removed when the age is less than 10 Myr. Viscosity of elements on the ocean-side will turn into weak zone with a decreased viscosity *η_weakzone_* to 1% or 10% of the original value to enable automatic generation of oceanic subduction. b, Elements in weak zones are identified by their distance *d_min_* from the special tracers, i.e., *d_edge_* < *d_min_* < *d_edge_* + 200 km.

Table S1. Physical parameters and their values.

| Parameter | Value^a^ |
| --- | --- |
| Earth radius *R* | 6371 km |
| Mantle thickness *d* | 2870 km |
| Surface thermal expansivity *α*_0_ | 3×10^-5^ K^-1^ |
| Thermal diffusivity *κ* | 1×10^-6^ m^2^ s^-1^ |
| Thermal conductivity *k* | 4.08 W m^-1^ K^-1^ |
| Mantle density *ρ* | 3400 kg m^-3^ |
| Specific heat *C_p_* | 1200 J kg^-1^ K^-1^ |
| Gravitational acceleration *g* | 10 m s^-2^ |
| Activation energy *E* | 8 (200 kJ mol^-1^) |
| Mantle reference temperature *T_r_* | 0.48 (1713 K) |
| Temperature offset *T_o_* | 0.5 (1773 K) |
| Internal heating *H* | 85 (7.84×10^-12^ W kg^-1^) or 114 (10.5×10^-12^ W kg^-1^) |
| Surface temperature *T_s_* | 273 K |
| Reference viscosity *η_ref_* | 8×10^21^ Pa·s |
| Effective dissipation number *Di_effective_* | 0.74 |
| Pre-factor *η_r_*(*z*) | $\left\{ \begin{aligned} 1, &z*R<100 \text{km} \\ 0.01, &100 \text{km}\leq z*R<\text{410 km} \\ 0.03, &410 \text{km}\leq z*R<660 \text{km} \\ 5.71z+0.43, &z*R\geq660 \text{km} \end{aligned} \right.$ |
| Pre-factor *η_c_*(*C_i_*) | $\left\{ \begin{aligned} 100, &i=1,\cdots5 \text{continents} \\ 5, &i=6 \text{orogens} \\ 1, &i=7 \text{mantle bottom thermo-chemical layer} \end{aligned} \right.$ |
| Yield stress σ*_y_*(*C_i_*) | $\left\{ \begin{aligned} 125, 150, \text{or} 175 \text{MPa}, &i=0 \text{oceanic lithosphere} \\ 400 \text{MPa}, &i=1,\cdots,5 \text{continents} \\ 50 \text{MPa}, &i=6 \text{orogens} \end{aligned} \right.$ |
| Chemical extra density *Δρ_c_i_* | $\left\{ \begin{aligned} -200 \text{kg m}^{-3}, &i=1,\cdots,5 \mathrm{continents} \\ -200 \text{kg m}^{-3}, &i=6 \mathrm{orogens} \\ 15, 30, \text{or} 60 \text{kg m}^{-3}, &i=7 mantle bottom \\ \mathrm{thermo}\text{-}\mathrm{chemical}\mathrm{layer} \end{aligned} \right.$ |
| Temperature contrast between surface and the CMB Δ*T* | 3000 K |
| Temperature contrast between surface and the asthenosphere Δ*T_lith_* | 1300 K |
| Thickness of continental lithosphere | 200 km |
| Thickness of orogens | 80 km |
| Initial thickness of the lower mantle thermo-chemical layer | 100, 250, or 400 km |
| Maximum viscosity cutoff | 1500 |
| Minimum viscosity cutoff | 0.01 |

^a^ The true values corresponding to the non-dimensional parameters are given in the parentheses right following.

Table S2. Tested cases.

| Case No. | Yield stress of oceanic lithosphere σ*_y_*(*C_0_*) (MPa) | *D_olith_* (km) | *H* | Initial thickness of the lower mantle thermo-chemical layer (km) | Extra density of the lower mantle thermo-chemical layer *Δρ_c_7_* (kg m^-3^) | *D_crust_* (km)^a^ | *η*_weakzone_ | Supercontinent assembly type |
| --- | --- | --- | --- | --- | --- | --- | --- | --- |
| 1 | 125 | 100 | 85 | 250 | 30 | – | 0.01 | Extroversion |
| 2 | 150 | 100 | 85 | 250 | 30 | – | 0.01 | Transitional |
| 3 | 175 | 100 | 85 | 250 | 30 | – | 0.01 | Introversion |
| 4 | 125 | 100 | 85 | 400 | 30 | – | 0.01 | Extroversion |
| 5 | 125 | 100 | 85 | 100 | 30 | – | 0.01 | Extroversion |
| 6 | 125 | 100 | 85 | 250 | 60 | – | 0.01 | Extroversion |
| 7 | 125 | 100 | 85 | 250 | 15 | – | 0.01 | Extroversion |
| 8 | 175 | 100 | 85 | 400 | 30 | – | 0.01 | Introversion |
| 9 | 175 | 100 | 85 | 100 | 30 | – | 0.01 | Introversion |
| 10 | 175 | 100 | 85 | 250 | 60 | – | 0.01 | Introversion |
| 11 | 175 | 100 | 85 | 250 | 15 | – | 0.01 | Introversion |
| 12 | 125 | 100 | 114 | 250 | 30 | – | 0.01 | Extroversion |
| 13 | 175 | 100 | 114 | 250 | 30 | – | 0.01 | Introversion |
| 14 | 125 | 100 | 85 | 250 | 30 | – | 0.1 | Extroversion |
| 15 | 175 | 100 | 85 | 250 | 30 | – | 0.1 | Introversion |
| 16 | 125 | 60 | 85 | 250 | 30 | – | 0.01 | Extroversion |
| 17 | 175 | 60 | 85 | 250 | 30 | – | 0.01 | Introversion |
| 18 | 125 | 100 | 85 | 250 | 30 | 15 | 0.01 | Extroversion |
| 19 | 125 | 100 | 85 | 250 | 30 | 20 | 0.01 | Transitional |
| 20 | 125 | 100 | 85 | 250 | 30 | 25 | 0.01 | Introversion |

^a^ ‘–’ means the plastic yielding in crust has a constant value same as the other parts of oceanic lithosphere. *D_olith_* = thickness of the oceanic lithosphere; *H* = non-dimensional mantle internal heating rate; *D_crust_* = thickness of the oceanic crust; *η*_weakzone_ = viscosity drop in weak zones.

Movie S1. Animations of extroversion supercontinent assembly (Case 1). Animations a and b show the mantle interior structures in 3-D spherical geometry with view centers at longitude/latitude = 0°/0° and 0°/–90°, respectively. Yellow and blue regions show the 0.1 and –0.1 (–0.05 for the top 300 km) non-dimensional residual temperature contours, marking mantle upwellings and downwellings, respectively. The semi-transparent gray denotes the continents, whereas the red regions show the lower mantle thermos-chemical layer. The residual temperature along cross section XYZ in animation c (blue, yellow, and orange) is shown together with 3-D presentation of the lower mantle thermos-chemical layer (red), in which the dark-red region denotes the CMB. Animation d shows the surface viscosity field of the model. Animation e shows the topographic contours of the lower mantle thermos-chemical layer above the CMB (warm colors) and the subducted slabs at various depths (cold colors).

Movie S2. Animation of transitional supercontinent assembly model (Case 2). See caption for Movie S1 for details.

**Movie S3**. Animations of introversion supercontinent assembly model (Case 3). See caption for Movie S1 for details.

References for Supplementary Data

1. King SD, Lee C and Van Keken PE *et al.* A community benchmark for 2-D Cartesian compressible convection in the Earth’s mantle. *Geophys J Int* 2010; **180**: 73–87.

2. Hansen U and Yuen DA. Effects of depth-dependent thermal expansivity on the interaction of thermal-chemical plumes with a compositional boundary. *Phys Earth Planet Inter* 1994; **86**: 205–21.

3. Chopelas A and Boehler R. Thermal expansion measurements at very high pressure, systematics, and a case for a chemically homogeneous mantle. *Geophys Res Lett* 1989; **16**: 1347–50.

4. Čížková H, van den Berg A and Jacobs M. Impact of compressibility on heat transport characteristics of large terrestrial planets. *Phys Earth Planet Inter* 2017; **268**: 65–77.

5. Tackley PJ. Self-consistent generation of tectonic plates in time-dependent, three-dimensional mantle convection simulations 1. Pseudoplastic yielding. *Geochem Geophys Geosyst* 2000; **1**: 2000GC000036.

6. Zhong S, Zhang N and Li Z-X *et al.* Supercontinent cycles, true polar wander, and very long-wavelength mantle convection. *Earth Planet Sci Lett* 2007; **261**: 551–64.

7. Li Z-X and Zhong S. Supercontinent-superplume coupling, true polar wander and plume mobility: Plate dominance in whole-mantle tectonics. *Phys Earth Planet Inter* 2009; **176**: 143–56.

8. Burke K and Torsvik TH. Derivation of Large Igneous Provinces of the past 200 million years from long-term heterogeneities in the deep mantle. *Earth Planet Sci Lett* 2004; **227**: 531–8.

9. Lowman JP and Jarvis GT. Mantle convection models of continental collision and breakup incorporating finite thickness plates. *Phys Earth Planet Inter* 1995; **88**: 53–68.

10. Huang C, Zhang N and Li Z-X *et al.* Modeling the Inception of Supercontinent Breakup: Stress State and the Importance of Orogens. *Geochem Geophys Geosyst* 2019; **20**: 1–19.

11. Kohlstedt DL, Evans B and Mackwell SJ. Strength of the lithosphere: Constraints imposed by laboratory experiments. *J Geophys Res Solid Earth* 1995; **100**: 17587–602.

12. Hyndman RD, Currie CA and Mazzotti SP. Subduction zone backarcs, mobile belts, and orogenic heat. *GSA Today* 2005; **15**: 4–10.

13. Mei S, Suzuki AM and Kohlstedt DL *et al.* Experimental constraints on the strength of the lithospheric mantle. *J Geophys Res Solid Earth* 2010; **115**: B08204.

14. Zhong S and Watts AB. Lithospheric deformation induced by loading of the Hawaiian Islands and its implications for mantle rheology. *J Geophys Res Solid Earth* 2013; **118**: 6025–48.

15. Jain C, Korenaga J and Karato S. On the Yield Strength of Oceanic Lithosphere. *Geophys Res Lett* 2017; **44**: 9716–22.

16. Moresi L and Solomatov V. Mantle convection with a brittle lithosphere: thoughts on the global tectonic styles of the Earth and Venus. *Geophys J Int* 1998; **133**: 669–82.

17. Yoshida M. Effects of various lithospheric yield stresses and different mantle-heating modes on the breakup of the Pangea supercontinent. *Geophys Res Lett* 2014; **41**: 3060–7.

18. Mallard C, Coltice N and Seton M *et al.* Subduction controls the distribution and fragmentation of Earth’s tectonic plates. *Nature* 2016; **535**: 140–3.

19. Burov EB. Rheology and strength of the lithosphere. *Mar Pet Geol* 2011; **28**: 1402–43.

20. Byerlee J. Friction of rocks. *Pure Appl Geophys* 1978; **116**: 615–26.

21. Boettcher MS, Hirth G and Evans B. Olivine friction at the base of oceanic seismogenic zones. *J Geophys Res Solid Earth* 2007; **112**: B01205.

22. Rolf T, Coltice N and Tackley PJ. Linking continental drift, plate tectonics and the thermal state of the Earth’s mantle. *Earth Planet Sci Lett* 2012; **351–352**: 134–46.

23. Gurnis M. Large-scale mantle convection and the aggregation and dispersal of supercontinents. *Nature* 1988; **332**: 695–9.

24. Yoshida M. Mantle temperature under drifting deformable continents during the supercontinent cycle. *Geophys Res Lett* 2013; **40**: 681–6.

25. Dang Z, Zhang N and Li Z-X *et al.* Weak orogenic lithosphere guides the pattern of plume-triggered supercontinent break-up. *Commun Earth Environ* 2020; **1**: 51.
